# Crystal Structure of the Heteromolecular Chaperone, AscE-AscG, from the Type III Secretion System in *Aeromonas hydrophila*

**DOI:** 10.1371/journal.pone.0019208

**Published:** 2011-04-29

**Authors:** Chiradip Chatterjee, Sundramurthy Kumar, Smarajit Chakraborty, Yih Wan Tan, Ka Yin Leung, J. Sivaraman, Yu-Keung Mok

**Affiliations:** Department of Biological Sciences, National University of Singapore, Singapore; Auburn University, United States of America

## Abstract

**Background:**

The putative needle complex subunit AscF forms a ternary complex with the chaperones AscE and AscG in the type III secretion system of *Aeromonas hydrophila* so as to avoid premature assembly. Previously, we demonstrated that the C-terminal region of AscG (residues 62–116) in the hetero-molecular chaperone, AscE-AscG, is disordered and susceptible to limited protease digestion.

**Methodology/Principal Findings:**

Here, we report the crystal structure of the ordered AscG_1–61_ region in complex with AscE at 2.4 Å resolution. Helices α2 and α3 of AscE in the AscE-AscG_1–61_ complex assumes a helix-turn-helix conformation in an anti-parallel fashion similar to that in apo AscE. However, in the presence of AscG, an additional N-terminal helix α1 in AscE (residues 4–12) is observed. PscG or YscG in the crystal structures of PscE-PscF-PscG or YscE-YscF-YscG, respectively, assumes a typical tetratricopeptide repeat (TPR) fold with three TPR repeats and one C-terminal capping helix. By comparison, AscG in AscE-AscG_1–61_ comprises three anti-parallel helices that resembles the N-terminal TPR repeats in the corresponding region of PscG or YscG in PscE-PscF-PscG or YscE-YscF-YscG. Thermal denaturation of AscE-AscG and AscE-AscG_1–61_ complexes demonstrates that the C-terminal disordered region does not contribute to the thermal stability of the overall complex.

**Conclusion/Significance:**

The N-terminal region of the AscG in the AscE-AscG complex is ordered and assumes a structure similar to those in the corresponding regions of PscE-PscG-PscF or YscE-YscF-YscG complexes. While the C-terminal region of AscG in the AscE-AscG complex is disordered and will assume its structure only in the presence of the substrate AscF. We hypothesize that AscE act as a chaperone of the chaperone to keep AscG in a stable but partially disordered state for interaction with AscF.

## Introduction

*Aeromonas hydrophila* is a ubiquitous Gram-negative bacterium that often leads to motile aeromonad septicemia in both fish and human [1], [2], characterized by gastroenteritis, wound infections and systemic illness [3]. Many Gram-negative bacteria exploit host cellular functions through the use of type III secretion systems (T3SSs) for host penetration and effector delivery [4]. The T3SS is complex, comprising more than 20 proteins spanning three membranes, which ensure the successful delivery of effectors [5], [6], [7]. Recent insight into this complexity has been gained by the understating of the intricate structures of the inner and outer membrane rings, the associated ATPase, the needle complex, and the interaction of chaperones and substrates of T3SS. A T3SS gene cluster has been located in *A. hydrophila* AH-1 and shown to be necessary for its pathogenesis [8]. At least three T3SS-secreted proteins (or effector proteins) have been identified in the extracellular proteome of a T3SS-negative regulator mutant but not in a T3SS-deficient mutant [9]. One of these effector proteins showed homology to AexT/AexU effector which has been reported recently in *A. hydrophila* strains AH-3 [10] and SSU [11], [12].

Chaperone proteins are required to avoid premature oligomerization of the needle complex subunit or translocators, and to maintain effectors in a form ready to be translocated in the T3SS system. These chaperones keep the subunit in a soluble and monomeric form inside the bacterial cell. There are several key examples identified over the last decade that demonstrate the importance of chaperones. For instance, the dimeric class I chaperone, SycE, maintains the non-native conformation of the effector, YopE, in *Yersinia pseudotuberculosis* [13]. In *Pseudomonas aeruginosa*, PscE and PscG interact to form a heteromolecular chaperone, PscE-PscG, which traps the needle complex subunit, PscF, in a monomeric state by forming a 1∶1∶1 ternary PscE-PscF-PscG complex. When the proteins were expressed and purified separately, neither PscE nor PscG could rescue polymerized PscF [14]. The dimeric class II chaperone LcrH/SycD consists of three tetratricopeptide repeat (TPR)-like motifs and interacts separately with translocators YopB and YopD in *Y. pseudotuberculosis* [15]. More recently, crystal structures of SycD from *Y. enterocolitica* [16] and PcrH from *P. aeruginosa* in complex with a short peptide from PopD were determined [17].

The crystal structures of chaperones that are required for the needle-complex subunit, for example, AscE from *A. hydrophila* (PDB ID: 2Q1K) [18] and YscE from *Y. pestis* (PDB ID: 1ZW0) [19], have revealed that both dimeric proteins comprise two helix-turn-helix monomers packed in an anti-parallel fashion. The recent crystal structure of the YscE-YscF-YscG complex (PDB ID: 2P58) demonstrated that YscE interacts with the N-terminal TPR motif of YscG. YscG binds tightly to the C-terminal half of YscF which adopts an α-helical hairpin conformation [20]. The analogous crystal structure of the PscE-PscF^55–85^-PscG complex (PDB ID: 2UWJ) revealed that the PscE-PscG heterodimeric chaperone folded in the form of a cupped hand with the C-terminus of PscF engulfed within the hydrophobic groove of PscG [21]. In both cases, the substrate adopted a non-native conformation, and PscF and YscF substrates were disordered at the N-terminus.

Other than the needle-complex subunit, the effector and translocator display disordered regions when in complex with their respective chaperone. For instance, the S1 region of the effector YopE remained disordered in the presence or absence of the chaperone SycE [22]. Moreover, we have shown that large regions of the translocators AopB and AopD were disordered and susceptible to limited protease digestion when in complex with the chaperone AcrH [23]. It seems that the presence of disordered regions in the substrate is a common characteristic in the chaperone-substrate complex of T3SS. However, the chaperone itself typically does not contain any disordered regions, such as the chaperone YscE for effector and the chaperone AcrH for translocators. Interestingly, our previous work contrasts this; we found that the C-terminal region (residues 62–116) of the chaperone AscG is disordered when in complex with AscE, while the N-terminal 61 residues of AscG in the AscE-AscG complex is resistant to protease digestion [23].

Here, we report the crystal structure of the heteromolecular chaperone formed by AscE and the N-terminal 61 residues of AscG from *A. hydrophila* AH-1 (PDB ID: 3PH0) refined to 2.4 Å resolution. Notably, we found that this N-terminal region of AscG assumed a conformation identical to the corresponding regions in the PscE-PscF-PscG or YscE-YscFG complexes. Taking into consideration previous work in the field, we propose that AscE is required to keep the N-terminal region of AscG in a stable and ordered conformation, while the disordered C-terminal region of AscG will only be induced to fold upon interaction with the AscF substrate.

## Results

### Overall structure of AscEG_1–61_ complex

**Figure 1:**
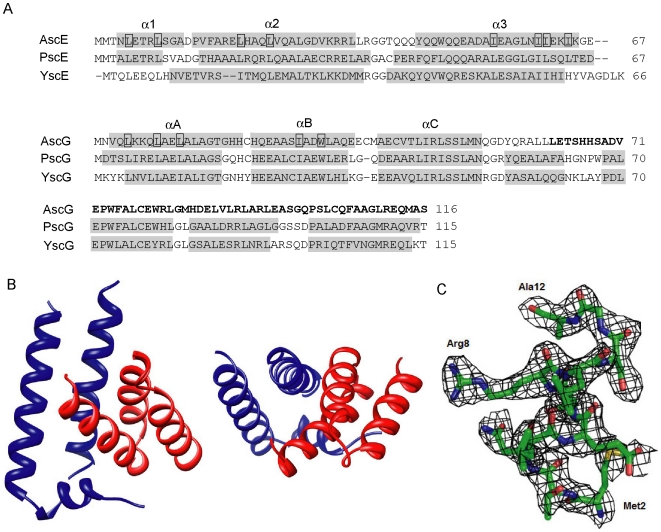
Sequence alignment and structure of the AscE-AscG_1–61_ complex. (A) Protein sequences of AscE and AscG from *Aeromonas hydrophila* AH-1 were aligned with related proteins from *Pseudomonas aeruginosa* (PscE and PscG) and *Yersinia pestis* (YscE and YscG) using CLUSTLAW [43]. The α-helical secondary structures, as determined in the crystal structures of AscE-AscG_1–61_, PscE-PscF-PscG and YscE-YscF-YscG, are shaded in grey. The residues involved in forming the hydrophobic interface between AscE and AscG_1–61_ are boxed. Residues 62 to 116 from the C-terminal disordered region of AscG are bold-faced. (B) Ribbon representations of the crystal structure of the complex formed between AscE (blue) and AscG_1–61_ (red) at two different views. The ribbon diagrams were generated using the software Chimera [44]. (C) The simulated-annealing *F_o_-F_c_* omit map in the conserved region of AscE in the AscE-AscG_1–61_ complex. The map is contoured at a level of 2.0 σ. Residues Met2 to Ala12 of AscE and all atoms within 2 Å of Met2 to Ala12 of AscE were omitted prior to refinement. The figure was generated with the graphics programs “PyMOL” [45].

The structure of AscE-AscG_1–61_ was solved by molecular replacement method and refined to a final R-factor of 0.239 (R_free_ = 0.292) at 2.4 Å resolution. The model was refined with good stereochemical parameters (Table 1 and Fig. 1C). Each asymmetric unit contained two molecules of AscE-AscG_1–61_ complex. Interpretable electron density was not observed for the residues in the loop region (residues 37–40) between helices α2 and α3 and the C-terminal residues Gly54 to Leu61. The helices α2 (residues 14–35) and α3 (residues 44–65) of AscE formed a pair of anti-parallel helices in the AscE-AscG_1–61_ complex with a topology similar to those in the crystal structure of apo AscE [18] (Fig. 1B). The N-terminal 14 residues of AscE were disordered in the crystal structure of apo AscE. In contrast, when in complex with AscG_1–61_, this region was induced to form a two-turn helix, α1 (residues 4–12), located in a perpendicular orientation to α2, similar to that which is observed by PscE in the PscE-PscF-PscG complex [21] (Fig. 1B). Within the AscE-AscG_1–61_ complex, the helices α1 and α2 of AscE only interacted with helix αA of AscG_1–61_, while helix α3 of AscE interacted with both helices αA and αB of AscG_1–61_ (Fig. 1B).

**Table 1:** Crystallographic data and Refinement Statistics.

Data set	Peak	Inflection	Remote	Native
**Data collection**				
Resolution range (Å)	50.0 - 2.8	50.0-2.8	50.0-2.8	50.0-2.4
Wavelength (Å)	0.9792	0.9794	0.9640	1.542
Observed reflections	20476	20196	20322	13757
Unique reflections	11163	11007	11087	7607
Completeness (%)	99.8	99.8	99.8	99.8
Overall *(I/σI)*	7.6	8.6	8.9	10.3
R_sym_ a (%)	8.9	11.1	8.4	10.1
**Refinement and quality**				
Resolution range (Å) I>σ2(I)				20.0 - 2.4
R_work_ b (no. of reflections)				0.239 (15264)
R_free_ c (no. of reflections)				0.292 (1601)
RMSD bond lengths (Å)				0.007
RMSD bond angles(deg)				1.40
**Average B-factors (Å^2^)**				
Main chain atoms				48.18
Side chain atoms				51.82
**Ramachandran plot**				
Most favored regions (%)				93.1
Additional allowed regions (%)				5.1
Generously allowed regions (%)				1.0
Disallowed regions (%)				0.0

a R_sym_ = |I_i_−<I>|/|I_i_| where I_i_ is the intensity of the i^th^ measurement, and <I> is the mean intensity for that reflection.

b R_work_ = |F_obs_−F_calc_|/|F_obs_| where F_calc_ and F_obs_ are the calculated and observed structure factor amplitudes respectively.

c R_free_ = as for R_work_, but for 10.0% of the total reflections chosen at random and omitted from refinement.

### Sequence and structural homology

The sequence identity between PscE and YscE is only 25.4%. However, the sequence alignment of AscE-AscG to PscE-PscF-PscG and YscE-YscF-YscG revealed a 43.3% and 38.8% sequence homology, respectively. AscG_1–61_ has a 55.7% sequence identity to the corresponding regions in both PscG and YscG, but the C-terminal disordered region of AscG is less conserved, with sequence identities of only 45.5% and 41.8% to PscG and YscG, respectively (Fig. 1A).

The structure of PscG or YscG in the PscE-PscF-PscG or YscE-YscF-YscG complexes comprised three tetratricopeptide repeats (TPR) and an additional C-terminus α-helix, forming a palm-shaped molecule [20], [21]. Here, we showed that the structure of AscG_1–61_ in the AscE-AscG_1–61_ complex consisted of an anti-parallel three helices bundle, with topology similar to the first on-and-a-half of the N-terminal TPR of PscG/YscG in both PscE-PscF-PscG/YscE-YscF-YscG complexes. The remaining one-and-a-half TPR and α-helix in the C-terminus was disordered in the AscE-AscG complex. Furthermore, in the AscE-AscG_1–61_ complex, only residues from the first TPR (αA, residues 3–20; and αB, residues 22–35) interacted with AscE, whereas the helix αC (residues 39–52) from the N-terminal half of the second TPR showed no points of contact with AscE (Fig. 1B).

**Figure 2:**
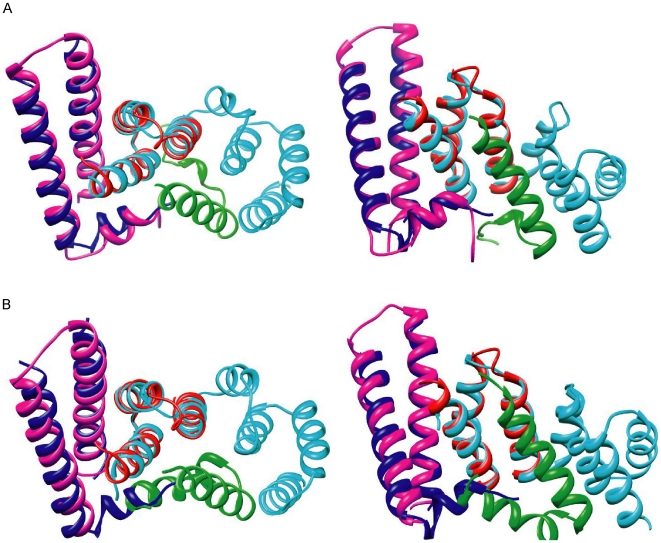
Superposition of the structure AscE-AscG_1–61_ with PscE-PscF-PscG and YscE-YscF-YscG. The crystal structure of AscE-AscG_1–61_ was superpositioned with (A) PscE-PscF-PscG or (B) YscE-YscF-YscG at two different views. The AscE and AscG_1–61_ proteins are colored blue and red, respectively. The E, F and G proteins in the PscE-PscF-PscG or YscE-YscF-YscG complexes are colored magenta, green and cyan, respectively.

Previously, it was demonstrated that the major difference between the crystal structures of PscE-PscG and YscE-YscG, aside from the additional α-helix at the N-terminus of PscE, was the position of the two α-helices of the E protein relative to the G protein [20]. Using the program “TopMatch” [24], [25], superposition of PscE with YscE in PscE-PscF-PscG and YscE-YscF-YscG, respectively, had a root mean square deviation (rmsd) of 0.7 Å, while superposition of PscG with YscG in PscE-PscF-PscG and YscE-YscF-YscG, respectively, had a rmsd of 1.1 Å. However, overlay of the full structures of PscE-PscG and YscE-YscG showed a relatively higher rmsd of 1.7 Å, consistent with the above observation. Similarly, the structure of AscE-AscG_1-61_ was superpositioned and compared with the corresponding regions in PscE-PscF-PscG and YscE-YscF-YscG (Fig. 2). We identified that superposition of AscE-AscG_1–61_ had a rmsd of 1.1 Å with either PscE-PscF-PscG or YscE-YscF-YscG, suggesting that the orientation of AscE relative to AscG lies intermediate between PscE-PscF-PscG and YscE-YscF-YscG. The individual molecules of AscE and AscG_1–61_ showed a similar structure to the corresponding regions in the PscE-PscF-PscG and YscE-YscF-YscG complexes. Superposition of AscE with PscE and YscE from each of the respective complexes had rmsd of 1.2 Å and 0.8 Å, respectively, whereas this was reduced with superposition of AscG_1–61_ with PscG and YscG at 0.8 Å and 0.9 Å, respectively.

### Residues at interfaces of the AscG_1–61_ complex

**Figure 3:**
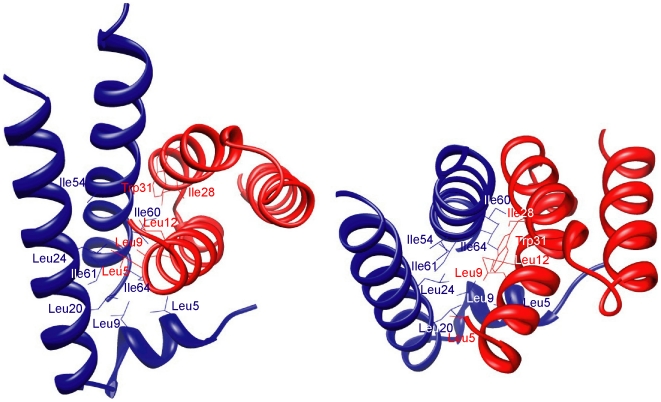
Residues at the hydrophobic interface between AscE and AscG_1–61_. Ribbon representation of the AscE-AscG_1–61_ crystal structure showing side chains of residues involved in the hydrophobic interface between AscE (blue) and AscG_1–61_ (red), at two different views.

Using the web server PISA (Protein interfaces, surfaces and assemblies) at the European Bioinformatics Institute (http://www.ebi.ac.uk/msd-srv/prot_int/pistart.html) [26], the surface area buried at the interface between AscE and AscG_1–61_ was determined to be 893.2 Å^2^ for each molecule. This buried area accounts for 20% of the total surface area of AscE (4591.1 Å^2^) and is predominantly hydrophobic in nature. Vicinity search based on the structure of AscE-AscG_1–61_ showed that residues from all three helices of AscE interacted with AscG. AscE residues Leu5 and Leu9 on helix α1; Leu20 and Leu24 on helix α2; and Ile54, Ile60, Ile61 and Ile64 on helix α3 formed a hydrophobic core between AscE and AscG_1–61_ through their interaction with AscG_1–61_ (Fig. 3). Several of these hydrophobic residues (Leu20, Leu24, Ile60 and Ile64) interacted with the respective G molecule in the crystal structures of both PscE-PscF-PscG and YscE-YscF-YscG. Leu5 and Leu9 were only conserved in PscE-PscF-PscG as interacting residues, as the N-terminal helix α1 was not observed in YscE-YscF-YscG, and Ile54 and Ile61 were conserved in YscE-YscF-YscG as interacting residues but not in PscE-PscF-PscG. For the contribution provided by AscG_1–61_, only residues from helices αA and αB of the first TPR interacted with AscE in the AscE-AscG_1–61_ complex. Leu5, Leu9 and Leu12 from helix αA, and Ile28 and Trp31 from helix αB of AscG_1–61_ were involved in forming the hydrophobic core with AscE (Fig. 3). By comparison, in the crystal structure of PscE-PscF-PscG and YscE-YscF-YscG, Leu5, Leu9 and Leu12 were also conserved interacting residues, while residues Ile28 and Trp31 were reported as interacting residues only in YscE-YscF-YscG. Interestingly, some of the reported interacting hydrophobic residues in the crystal structures of PscE-PscF-PscG and YscE-YscF-YscG were replaced with charged or polar residues in AscE or AscG, indicating that these residues may not be essential for the interaction. For instance, Ile6 and Leu35 of PscG were replaced by Lys6 and Glu35 in AscG; Val43 and Ala54 of YscE were replaced by Gln46 and Gly57 in AscE; and Leu8 and Ile15 of YscG were replaced by Gln8 and Ala15 in AscG.

**Figure 4:**
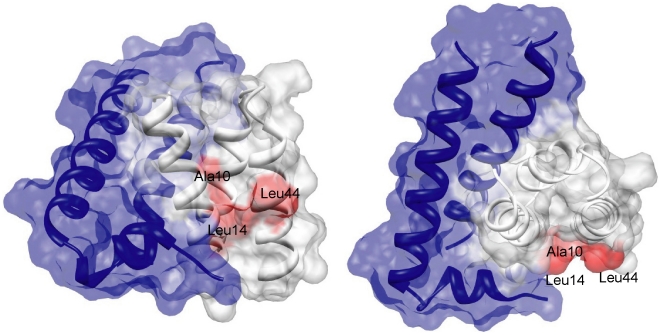
Surface diagram of the AscE-AscG_1–61_ complex and predicted AscF interacting residues. Surface diagram of the AscE-AscG_1–61_ complex showing the tight interaction between AscE (blue) and AscG_1–61_ (white), at two different views. The residues on AscG_1–61_ predicted to interact with AscF based on the crystal structures of PscE-PscF-PscG and YscE-YscF-YscG are colored red. The surface diagrams were generated using the software Chimera [44].

Based on the structure of PscE-PscF-PscG and the residues on PscG reported to interact with PscF, we predict that the corresponding residues on AscG_1–61_ will interact with AscF: Ala10 and Leu14 on helix αA; Leu32 on helix αB; and Val42 and Leu44 on helix αC. In contrast, based on the structure of YscE-YscF-YscG, the corresponding residues of AscG_1–61_ are predicted to interact with AscF: Lys7, Ala10 and Leu14 on helix αA; and Cys41 and Leu44 on helix αC. We speculate that highly conserved residues, such as Ala10 and Leu14 on helix αA and Leu44 on helix αC on AscG_1–61_, are essential residues that should interact with AscF (Fig. 4). Other than the N-terminal ordered region (residues 1–61), most of the conserved residues on PscG/YscG reported to interact with PscF/YscF were located at the disordered region (residues 62–116) of AscG. For PscG, residues that correspond to the disordered region of AscG (Asp70, Pro73, Trp74, Leu77, Trp80, Ser102, Phe106 and Leu110) interacted with PscF in the PscE-PscF-PscG complex. Similarly, for YscG in the YscE-YscF-YscG complex, the main binding site for YscF is located from the 3^rd^ to 7^th^ helices includes almost identical residues corresponding to the disordered region of AscG (Val71, Pro73, Trp74, Leu77, Trp80, Phe106 and Leu110 of AscG). We hypothesize that these residues that are common to both complexes (Pro73, Trp74, Leu77, Trp80, Phe106 and Leu110) are essential residues at the disordered region of AscG involved in its interaction with AscF.

### Thermal denaturation of AscE-AscG and AscE-AscF-AscG complexes

**Figure 5:**
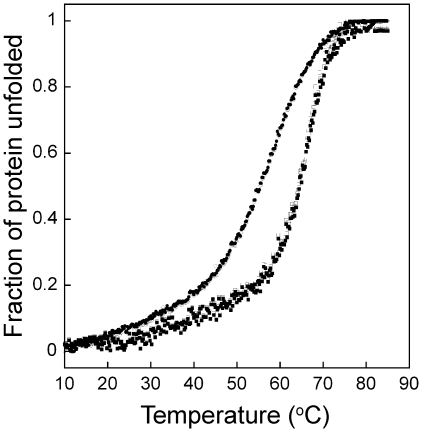
Thermal denaturation of AscE-AscG, AscE-AscG_1–61_, AscE-AscG-AscF and AscE-AscG-AscF_53–87_ complexes monitored by Far-UV CD. Thermal denaturation of the AscE-AscG_1–61_ (closed circle), AscE-AscG full length (open circle), AscE-AscG-AscF_53–87_ (closed square) and AscE-AscG-AscF full length (open square) complexes monitored by Far-UV CD at 220 nm from 10°C to 85°C.

To confirm the existence of disordered regions in AscE-AscG and AscE-AscF-AscG, we performed thermal denaturation monitored using Far-UV CD. It is assumed that the disordered region has a random coil conformation and should not contribute significantly to the thermal stability of the protein complex. Thermal denaturation experiments on AscE-AscG_1–61_ and AscE-AscG full length protein showed similar denaturation profiles and T_m_ of 66.5°C and 65.4°C, respectively (Fig. 5). The T_m_ was determined by curve fitting according to the equation by Ruiz-Sanz *et al.* [27]. These data suggested that the additional region of AscG in the AscE-AscG complex (residues 62–116) did not contribute to the overall thermal stability of the complex and likely assumed a disordered conformation. To further confirm this, we observed the thermal denaturations of AscE-AscG-AscF_53–87_ and AscE-AscG-AscF full length complexes. The results demonstrated T_m_ for denaturation of AscE-AscG-AscF_53–87_ and AscE-AscG-AscF complexes at 71.7°C and 71.1°C, respectively, indicating that residues 1–52 at the disordered region at the N-terminal region of AscF did not contribute to thermal stability of the whole complex (Fig. 5). We speculate that the overall higher thermal stability in the AscE-AscG-AscF complex as compared to the AscE-AscG complex is likely contributed by the C-terminal region of AscG, which becomes folded in the presence of AscF, as well as by the bound C-terminal region of AscF.

## Discussion

The chaperones in the T3SS of bacteria typically assume a dimeric form in solution. The class I chaperone YscE is a homodimer before and after binding to the effector YopE [13], [28]. In contrast, while the class II chaperone AcrH is also a homodimer before binding to the translocators AopB or AopD, it converts into a monomer in the complex [23]. The dimeric nature of the chaperones may be necessary to stabilize the protein in the absence of its substrate. The class III chaperone for the needle complex subunit is unique in that it is a heterodimer comprising E and G proteins. Previously, we discovered another unique feature of this class of chaperone: the presence of a disordered region in the G protein which only becomes folded in the presence of the substrate [18]. For the other two classes of chaperones, such a disordered region has not been reported, in either the absence or presence of the substrate.

In the T3SS system, the substrate, when in complex with the chaperone, usually attends a non-native conformation. For instance, the chaperone binding region of YopE wraps around YscE in a non-native conformation [13]. The chaperones, themselves, however, do not change their conformation after binding their substrate, but are maintained in a native conformation. In this work, we determined the crystal structure of the AscE-AscG_1–61_ complex to be surprisingly close to the native conformation of the corresponding region in the PscE-PscF-PscG or YscE-YscF-YscG complexes. The chaperone AscG maintains only half of the protein in a native conformation, while the other half is disordered and only becomes folded into a native conformation in the presence of substrate.

The structure of AscEG_1–61_, although similar to the corresponding region in PscE-PscF-PscG and YscE-YscF-YscG, still has noticeable differences when compared to these structures. The relative disposition of AscE and AscG in the complex is in between that of PscE-PscF-PscG and YscE-YscF-YscG. The N-terminal 14 residues of AscE, PscE and YscE are highly conserved (a major homology region) (Fig. 1A). Our previous work showed that the N-terminal 13 residues of the apo AscE dimer crystal structure were disordered and susceptible to limited protease digestion [18]. However, this region forms an additional α-helix only when AscE is in complex with AscG. This N-terminal α-helix is also found in PscE of the PscE-PscF-PscG complex, but the first 9 residues of YscE form part of a contiguous α-helix in the crystal structure of YscE [19] and becomes disordered in the YscE-YscF-YscG complex [20]. Others have reported that this N-terminal region is non-essential for the formation of the E-G-F complex, as this region can be removed and PscE can still form complex with G and F to give a functional T3SS [21]. Furthermore, it has been suggested that the N-terminal 14 residues of the E protein are not involved in binding with the substrate F, and in the PscE-PscF-PscG complex, only the residue Met2 of PscE interacts with PscF [21].

Although the N-terminal 14 residues of AscE may not be required to complex with G and F, AscE itself is essential to keep AscG soluble, monomeric, stable and with a disordered region to interact with AscF. Expression of AscG alone will go into inclusion bodies and the co-expression of AscG and AscF fails to form a complex [23]. AscG, or other class III chaperones such as YscG and PscG, assumes a typical TPR-like fold with seven helices (TPR 1–3 with a C-terminal capping helix). AcrH also contains a TPR fold with seven helices, but the sequence identity of the aligned region between AcrH and AscG is very low (22.4%). However, AcrH is stable and functional alone, while AscG requires AscE for stabilization and to keep it in a disordered state for functioning. The major difference between AcrH (class II chaperone) and AscG (class III chaperone) is that AcrH forms a dimer and contains extra residues at the region N-terminal to the TPR 1 that have the potential to form an additional helix.

IpgC is a class II chaperone of the T3SS in *Shigella* and each monomer consists of eight helices (H1–H8), with H1 (residues 9–21) situated N-terminal to TPR 1. IpgC forms an asymmetric dimer with H1 of chain A, which is stabilized by a hydrophobic interface provided by H1, H3, H4 and H5 of chain B. H1 and the loop connecting it to H2 show different arrangement in both subunits. If the first 20 residues is truncated, IpgC will form an aggregate with very high M.W. [29]. In *Yersinia*, the chaperone SycD forms a head-to-head dimer via TPR 1 (helices 1A and 1B), and formation of the dimer could stabilize the interface that may require AscE in the case of AscG. The region N-terminal to TPR 1 also form an additional helix h0 (residues 22–29), although it is not involved substantially in dimer formation [16]. The class II chaperone will only change from a dimer to a monomer when it is in complex with the translocator substrate [23]. Recent crystal structure of PcrH showed that it is also a dimer, although the dimer interactions are made through the convex side of the TPR fold instead of the N-terminal region and TPR 1. Also, there are no additional helices N-terminal to TPR 1 in PcrH as the N-terminal 20 residues are disordered and residues 21–31 are unstructured in the crystal structure [17].

Intrinsically unstructured proteins are more commonly found in eukaryotes than prokaryotes, and their role and functions have been reviewed extensively [30], [31]. Proteins that are intrinsically unstructured or contain extensive disordered regions are more malleable and can regulate and bind a diverse range of ligands. In addition, disordered proteins provide a larger intermolecular interface with a smaller protein size [30]. While these are advantages for cellular regulatory proteins, they are unlikely to be the reasons for AscG to have a disordered region, as AscF is its only known target. Instead, we believe that the presence of the disordered region in AscG enhances the speed of interaction [30], [32] between AscG and AscF, which may facilitate turnover and allow for the rapid assembly of the needle complex subunit of the T3SS. This use of disordered region to enhance the rate of interaction and uncouple specificity from binding affinity has been observed in the self-assembly processes of viruses and bacterial flagella, specifically during the process of regulating the addition of new components to the growing assembly [33], [34].

### Conclusions

In conclusion, we have shown the crystal structure of the class III chaperone, AscG, and demonstrated distinct differences in the organization of the protein: the N-terminus is ordered and natively folded under the help of another chaperone, AscE, whereas the C-terminal portion is disordered. This disordered half of the chaperone may facilitate fast turnover between itself and its binding substrate, AscF, with the presence of AscF, in turn, able to induce the chaperone to become ordered. As the sequence identity between the C-terminal regions of AscG with YscG and PscG are high, we expect that the corresponding regions in YscG and PscG are also disordered in the absence of their respective substrates, but, further investigations are warranted.

## Materials and Methods

### Cloning of AscE-AscG_1–61_

For co-expression of the complex formed between AscE and the N-terminal 61 residues of AscG (AscE-AscG_1–61_), full length AscE was sub-cloned at the second multiple-cloning site (MCS2) of the co-expression vector pETDuet-1 (Novagen) using NdeI and XhoI restriction enzymes. The pETDuet-1 vector encodes for ampicillin resistance. The truncated AscG was subsequently sub-cloned into the same vector at the first multiple-cloning site (MCS1), which contains the 6× His tag, using BamHI and EcoRI. This permitted co-expression of both proteins from the same plasmid. The correct constructs were confirmed by Big Dye DNA sequencing (Applied Biosystems).

### Expression and purification of AscE-AscG_1–61_

*Escherichia coli* BL21 (DE3) cells were transformed with the suitable plasmid and a single ampicillin-resistant colony was used to inoculate 50 ml of Luria Broth (LB) supplemented with 100 µg/ml of ampicillin. The culture was grown overnight at 37°C by shaking at 200 rpm. 10 ml of the overnight culture was used to inoculate 1 L of LB and the cells were grown to early log phase (OD_600_ = 0.6). Expression was induced with IPTG at a final concentration of 0.4 mM, and the cells were grown at 25°C for an additional 14 h, and then harvested by centrifugation (6,000 rpm, 15 min).

The cell pellet was resuspended in 30 ml of Ni-binding buffer (20 mM Tris-HCl pH 7.5, 500 mM NaCl, 5 mM imidazole) along with one tablet of Complete EDTA-free Cocktail Protease Inhibitor (Roche). Cells were lyzed using a sonicator and cell debris was removed by centrifugation (18,000 rpm, 30 min). The supernatant was purified using Ni-NTA beads (Qiagen). The proteins were eluted from the beads with Ni-binding buffer containing 0.5 M of imidazole, then dialyzed against 3 L of gel filtration buffer containing 20 mM Tris-HCl pH 7.5, 200 mM NaCl, 5% glycerol and 5 mM DTT. Proteins were further purified on a prepacked HiLoad 16/60 Superdex 75 prep grade (GE Healthcare) gel filtration column on the AKTA Fast Protein Liquid Chromatography system (GE Healthcare) using gel filtration buffer. Protein concentration was determined by UV absorption at 280 nm in a Hitachi spectrophotometer. Eluted proteins were stored at minus 80°C for subsequent analysis.

### Circular dichroism spectra and thermal denaturation

Circular dichroism (CD) measurements were performed room temperature using a J-810 spectropolarimeter (Jasco) equipped with a temperature-controlled sample holder. 300 µl sample of 25 µM protein in a buffer containing 20 mM Tris-HCl pH 7.5 and 5 mM DTT were used for thermal denaturation. Thermal denaturation was monitored by changes in CD ellipticity at 220 nm as a function of temperature from 10°C to 85°C with a heating rate of 2°C/min.

### Crystallization and structure determination

AscEG_1–61_ was dialyzed in a buffer containing 20 mM Tris-HCl pH 7.5 and 5 mM DTT, and concentrated to 12 mg/ml. Dynamic Light Scattering (DLS) was used to check the homogeneity of concentrated proteins. Subsequently, the Wizard crystal screen was carried out to identify the initial crystallization conditions. The diffraction quality crystals of Se-Met labeled AscEG_1–61_ (12 mg/mL in 10 mM Tris pH 7.4 and 5 mM DTT) was obtained at 25°C using a 1∶1 mixture of protein and 10% PEG (w/v) 3000, 0.1 M immidazole pH 8.0 and 0.2 M Li_2_SO_4_. Crystals belonged to space group P2_1_2_1_2 with unit cell dimensions a = 42.53, b = 71.15, and c = 86.42 Å. The asymmetric unit contained two complex molecules with 51.8% solvent. Crystals were cryo-protected in 20% glycerol, mounted in fiber loops, and flash cooled to 100 K (Oxford Cryostream). Diffraction data were collected at beamline 13B1 (National Synchroton Radiation Research Center, Taiwan) using an ADSC Quantum-315 CCD area detector. The native and anomalous data sets were indexed, integrated and scaled using the HKL 2000 suite of programs (Otwinowski and Minor, 1997). The structure was solved using Multiple Anomalous Diffraction (MAD) method. All the expected five selenium sites were located and refined by “HKL2map” (ShelX CDE) [35] and “BnP” [36] that resulted in an interpretable electron density map. Phases were further improved by iterative cycles of 4-fold non-crystallographic symmetry (NCS) averaging and solvent flattening with “DM” in “CCP4” programs suite [37]. The high resolution native data set collected in the in-house rotating anode generator was used to auto-build the initial model (∼50% residues) with “ARP/wARP” [38]; this traced the remaining chain as poly-alanine. Subsequently, the manual model building was completed using the programs “COOT” [39] and “O” [40] and alternated with refinement with the program “CNS” [41]. Model quality was verified with “PROCHECK” [42]. All residues were either in the most favored (93.1%) or additional allowed (5.1%) regions of the Ramachandran plot.

### Superposition of structures

For comparison of structures, the structure of AscE-AscG_1–61_ was superpositioned with the corresponding regions in PscE-PscF-PscG and YscE-YscF-YscG using “TopMatch” [24], [25] web service (http://topmatch.services.came.sbg.ac.at/TopMatchFlex.php). The root-mean-square error of superposition in Å was calculated using all structurally equivalent Cα atoms.

### Data deposition

The atomic coordinates of *A. hydrophila* AH-1 AscE-AscG_1–61_ have been deposited at the Protein Data Bank (PDB ID: 3PH0).
